# Comment on “Systematic Review and Meta‐Analysis of Protein Intake to Support Muscle Mass and Function in Healthy Adults” by Nunes et al.

**DOI:** 10.1002/jcsm.70036

**Published:** 2025-07-26

**Authors:** Omar Inca‐Barriga, Piero Alberti‐Nuñez, Miguel López‐Moreno

**Affiliations:** ^1^ Department of Nutrition and Dietetics Universidad Privada del Norte (UPN) Lima Perú; ^2^ Department of Nutrition and Dietetics Universidad Le Cordon Bleu (ULCB) Lima Perú; ^3^ Diet, Planetary Health and Performance, Faculty of Health Sciences Universidad Francisco de Vitoria Madrid Spain

We respectfully address you regarding the recently published article by Nunes et al., entitled ‘Systematic review and meta‐analysis of protein intake to support muscle mass and function in healthy adults’ [1]. The topic is highly relevant, and the study contributes valuable evidence to the field. However, we believe that certain methodological considerations may affect the interpretation and robustness of the conclusions drawn.

In the statistical analysis section, the authors state: ‘The analysis was conducted using change from baseline to immediate post‐treatment data (means, standard deviations) for both intervention and control/placebo groups to generate summary measures of effect in the form of standardized mean differences (SMDs)’. This implies that the SMDs were calculated based on within‐group pre‐post changes rather than on the between‐group differences at follow‐up. However, this approach has been explicitly discouraged by the Cochrane Handbook of Systematic Reviews of Interventions (v6.4, Section 10.5.2), which cautions:

Change‐from‐baseline outcomes may also be preferred if they have a less skewed distribution than post‐intervention outcome measurement. Although sometimes used as a device to ‘correct’ for unlucky randomization, this practice is not recommended [2].

This concern arises from the fact that pre‐post differences within a single group do not adequately control confounding factors and natural processes that affect all groups equally (such as the effects of training alone), potentially leading to artificially inflated effect sizes. Along these same lines, Cuijpers et al. note that calculating effect sizes from within‐group pre‐post comparisons without considering between‐group differences may lead to an overestimation of the intervention effect. In contrast, between‐group SMDs are more robust, as they better account for external variables and provide a more accurate estimate of the true effect of the intervention [3].

A particularly critical issue in the present meta‐analysis concerns the apparent inclusion of multiple intervention arms from the same study as independent entries in the subgroup analysis, with SMDs calculated from pre‐post differences within each arm. While this approach may allow for subgroup‐specific exploration (e.g., stratified by protein intake), it introduces a unit‐of‐analysis error if multiple arms from the same study are analysed independently without accounting for shared sources of variance. According to Cochrane Handbook guidelines (v6.4, Section 23.3.4), this practice violates the assumption of independence across comparisons and can artificially narrow confidence intervals, thereby inflating the precision of the summary effect size [2]. Given that no advanced statistical techniques (e.g., multivariate meta‐analysis) were employed to account for this dependence, the reported results likely overstate both the certainty and magnitude of the observed effects.

The analysis of the forest plot in Figure 2, which includes studies with protein intakes ≥ 1.6 g/kg/day, reveals that only four out of the 20 studies reported statistically significant effects. However, it is important to note that one of these studies—Burke et al.—found significant within‐group changes but did not report statistically significant between‐group differences [4]. Including this study in the analysis as if it had demonstrated superiority of the intervention group over placebo could have artificially contributed to the statistical significance of the subgroup, potentially leading to an overinterpretation of the intervention's efficacy at this protein intake threshold.

A relevant point to consider is that the study by Willoughby et al. presents outlier results favouring the intervention with dietary protein. In this study, the protein‐supplemented group also exhibited an increase in carbohydrate and fat intake, contributing an additional caloric intake of approximately 250 kcal, as evidenced by the greater change in body mass compared to the protein‐supplemented group [5]. A similar pattern is observed in other included studies, such as Nakayama et al. [6]. Furthermore, in the study by Oertzen‐Hagemann et al. [7], where caloric and macronutrient intake were not reported, a significant increase in body weight and lean mass was observed in the protein‐supplemented group compared to the placebo group. However, this change may have been influenced by a lack of control or adjustment of energy intake in both groups, making it difficult to attribute the effects solely to the intervention. Given that greater energy availability is a critical factor in lean mass gain [8], not adjusting for this variable limits the ability to isolate the effect of protein intake from the confounding impact of increased total energy intake. Although a sensitivity analysis excluding individual studies may be conducted, this approach is insufficient to fully address the issue.

Finally, we note with concern that the discussion paper states that ‘our data show a small increase in LBM caused by ingesting additional protein and RE.’ This statement implies a direct causal relationship that is not justified, particularly if the effect sizes were derived from within‐group analyses and if key confounding variables, such as total energy intake, were not adequately controlled in several included studies.

## Ethics Statement

The authors certify that they comply with the ethical guidelines for authorship and publishing in the *Journal of Cachexia, Sarcopenia and Muscle*.

## Conflicts of Interest

The authors declare no conflicts of interest.
